# Affordable optical clearing and immunolabelling in mouse brain slices

**DOI:** 10.1186/s13104-023-06511-y

**Published:** 2023-09-30

**Authors:** Phillip M. Muza, Marta Pérez, Suzanna Noy, Miyu Kurosawa, Loukia Katsouri, Victor L. J. Tybulewicz, Elizabeth M.C. Fisher, Steven J. West

**Affiliations:** 1 Department of Neuromuscular Diseases, Queen Square Institute of Neurology, University College London, Queen Square, London, WC1N 3BG UK; 2 Sainsbury Wellcome Centre, University College London, 25 Howland Street, London, W1T 4JG UK; 3 The Francis Crick Institute, London, NW1 1AT UK

**Keywords:** ABSOC, Calretinin, iDISCO, Immunolabeling, Optical Clearing

## Abstract

Traditional histological analysis is conducted on thin tissue sections, limiting the data capture from large tissue volumes to 2D profiles, and requiring stereological methods for 3D assessment. Recent advances in microscopical and tissue clearing methods have facilitated 3D reconstructions of tissue structure. However, staining of large tissue blocks remains a challenge, often requiring specialised and expensive equipment to clear and immunolabel tissue. Here, we present the Affordable Brain Slice Optical Clearing (ABSOC) method: a modified iDISCO protocol which enables clearing and immunolabeling of mouse brain slices up to 1 mm thick using inexpensive reagents and equipment, with no intensive expert training required. We illustrate the use of ABSOC in 1 mm C57BL/6J mouse coronal brain slices sectioned through the dorsal hippocampus and immunolabelled with an anti-calretinin antibody. The ABSOC method can be readily used for histological studies of mouse brain in order to move from the use of very thin tissue sections to large volumes of tissue – giving more representative analysis of biological samples, without the need for sampling of small regions only.

**Supplementary Information:**

The online version contains supplementary material available at 10.1186/s13104-023-06511-y.

## Introduction

Immunohistochemistry (IHC) has been long appreciated as an invaluable technique to explore biological tissue structure by visualising proteins and cells. Traditionally IHC has been performed on thin tissue sections to enable clear visualisation with widefield microscopy. Furthermore, thin sections provide easy access for consistent antibody labelling. However, the use of thin samples limits the imaging data to two-dimensions, providing a limited view of innately 3D structures. Stereological methods have been developed to assess 3D properties of tissues from 2D section data [1, 2], but their manual application and inability to fully capture 3D tissue structure have limited their usage.

The widespread emergence of optical sectioning methods in microscopy has made 3D reconstruction of tissue structure accessible. To fully utilise these techniques, histological methods for labelling and optically clearing tissues has become highly desirable. The recent re-discovery of optical clearing techniques in the last decade has met this need, enabling the analysis of 3D structures directly in tissues of interest [3].

Optical clearing methods can be divided into solvent-based methods, such as 3DISCO [4] and iDISCO [5], which produce high tissue transparency, but can impact on histological signals and induce substantial tissue shrinkage; or aqueous-based methods, such as CUBIC [6] and SeeDB [7], which can expand tissues, are prolonged in their application, and the resulting samples tend to retain histological signals well, yet are more limited in optical clarity than solvent-cleared tissues. Hydrogel embedding methods (CLARITY, PACT, and SWITCH) have been developed in an attempt to protect protein and tissue structure through the clearing process. However, these methods require specialist equipment and training, and whilst CLARITY is now commercially available, it is prohibitively expensive for many laboratories to undertake routinely [8–10].

To overcome these issues we have modified an iDISCO protocol for use in thick, 1 mm mouse brain slices. We call this new protocol the ABSOC (Affordable Brain Slice Optical Clearing) method. ABSOC is inexpensive and rapidly applied without the need for specialised equipment or expert training. We believe this method can be readily taken up by laboratories that currently use thin sections routinely, allowing the reconstruction of volumes of tissue to conduct 3D assessments of tissue structure. We illustrate the use of ABSOC in C57BL/6J mouse dorsal hippocampal and medial prefrontal cortex coronal brain slices of 1 mm in depth, immunolabeled with an anti-calretinin antibody.

## Main text

ABSOC is a straightforward and inexpensive method to optically clear and immunolabel thick brain slices, ready for imaging. Here, we describe the ABSOC procedure from brain fixation and storage, to optical clearing plus immunolabeling, using an anti-calretinin antibody as an example. We chose calretinin to illustrate our approach because this commercially available antibody produces a discrete signal in cell bodies and neuropil of positively-immunolabeled cells.

### Brain storage and sectioning

Wild-type (WT) mice from the B6;129S7-Dp(10Prmt2-Pdxk)2Yey/J strain colony, previously described in [11], were maintained at the Medical Research Council - Prion Unit at University College London and used in all experiments. Mice were housed in individually ventilated cages (IVC) of 2–5 age-matched animals under controlled environmental conditions (24–25 °C; 50–60% humidity; 12 h light/dark cycle) with free access to food and water. All experiments were performed under appropriate licences in accordance with the United Kingdom Animal (Scientific Procedures) Act 1986.

At 3-months age, WT mice were overdosed using the ‘drop jar’ method. Mice were placed in a 1 L jar containing a paper ball with 1 ml 100% isoflurane added. Mice rapidly lose consciousness and move through all four planes of anaesthesia until the sudden cessation of breathing. Death was then confirmed via exsanguination by transcardial perfusion. Overdose with isoflurane to cessation of breathing has several benefits, most importantly the dose is high enough to induce complete and permanent loss of all sensation, ensuring no welfare issues. The method is also very rapid, and the subsequent perfusion is performed more easily, quickly, and confidently as a result of complete loss of reflexes. Mice were initially transcardially perfused using 20 ml phosphate buffered saline (PBS) solution until the blood was clear and then 20 ml PBS + 4% formaldehyde prepared in PBS. Transcardial perfusion with PBS and PFA was performed to (1) remove blood as it interferes with optical clearing of tissue and (2) preserve and stabilise tissue before autolysis occurs. To confirm death, mice were decapitated and whole brains were extracted and post-fixed with 4% formaldehyde overnight at room temperature (RT), followed by storage with PBS + 0.05% sodium azide cooled to 4^o^C before use. PBS + 0.05% sodium azide is ideal for long-term storage of whole brains because of the bactericide properties of sodium azide, and we have routinely kept samples for 1 year in this medium.

When ready for analysis, 1 mm thick brain slices from the mice were sectioned. This was done using a 1 mm brain matrix (Kent Scientific – RBMS-200 C), where the whole brain was sectioned coronally at approximately Bregma 3.92 mm, -0.94 mm, and − 4.96 mm to produce three 4 mm tissue blocks (Kent Scientific – RBMS-200 C). Tissue was sectioned using a Vibratome (Leica VT1000S) to the thickness appropriate for the experiment. Medial prefrontal cortex tissue was sectioned from tissue blocks between Bregma 3.92 mm and − 0.94 mm; and dorsal hippocampal tissue was sectioned from tissue blocks between Bregma − 0.94 mm and − 5.56 mm.

### ABSOC optical clearing protocol and immunolabelling

A comprehensive list of reagents and safety precautions is described in Table 1. Reagents were chosen based on their affordability and optimal use in clearing tissue slices.

**Table 1:** Reagents and equipment used to immunolabel and optically clear ABSOC brain slices

Equipment and Reagents	Stock Code	Working Concentration	Safety Precautions
Adhesion Slides	VWR (630–0950)		
Amber Glass Vials	ThermoFisher (141-40ATS)		
Benzyl Alcohol	Sigma-Aldrich (305197)	33% v/v	Acute toxicity and flammable – store in flammable cabinet and handle in fume hood
Benzyl Benzoate	Sigma-Aldrich (B6630)	66% v/v	Acute toxicity, flammable, and hazardous to aquatic environment - store in a flammable cabinet, handle in fume hood, and dispose responsibly
Donkey anti-Goat AlexaFluor 488	Abcam (ab150129)	2 µg/ml	
Dichloromethane	Sigma-Aldrich (270997)	66% v/v	Acute toxicity and hazardous to health –handle in fume hood
Formaldehyde, Ultra Pure	PolySciences (Cat #04018-1)	10% v/v	Acute toxicity and hazardous to health –handle in fume hood
Goat anti-IBA1	Abcam (ab5076)	0.5 µg/ml	
Goat anti-Rabbit Fab fragments AlexaFluor 647	Jackson ImmunoResearch (111-607-008)	1.5 mg/ml	
Goat anti-Rabbit Secondary Antibody, AlexaFluor 633	Invitrogen (A-21071)	2 µg/ml	
Hydrogen Peroxide	Sigma-Aldrich (H1009)	5% v/v	Acute toxicity, corrosive, and flammable – store in a flammable cabinet and handle responsibly in a fume hood
Innova 44 Incubator Shaker	New Brunswick (M1282-0002)		
KS 260 Basic Shaker	IKA (#0002980202)		
Methanol	Sigma-Aldrich (34860)		Acute toxicity and flammable – store in flammable cabinet and handle in fume hood
Mouse brain matrix	Kent Scientific (RBMS-200C)		
Rabbit anti-Calretinin Antibody	Abcam (ab244299)	0.22 µg/ml	
Rabbit anti-GFAP Antibody	Abcam (ab7260)	1 µg/ml	
Rabbit anti-S100B Antibody	Abcam (ab52642)	1 µg/ml	
Vibratome	Leica (VT1000S)		

**Fig. 1:**
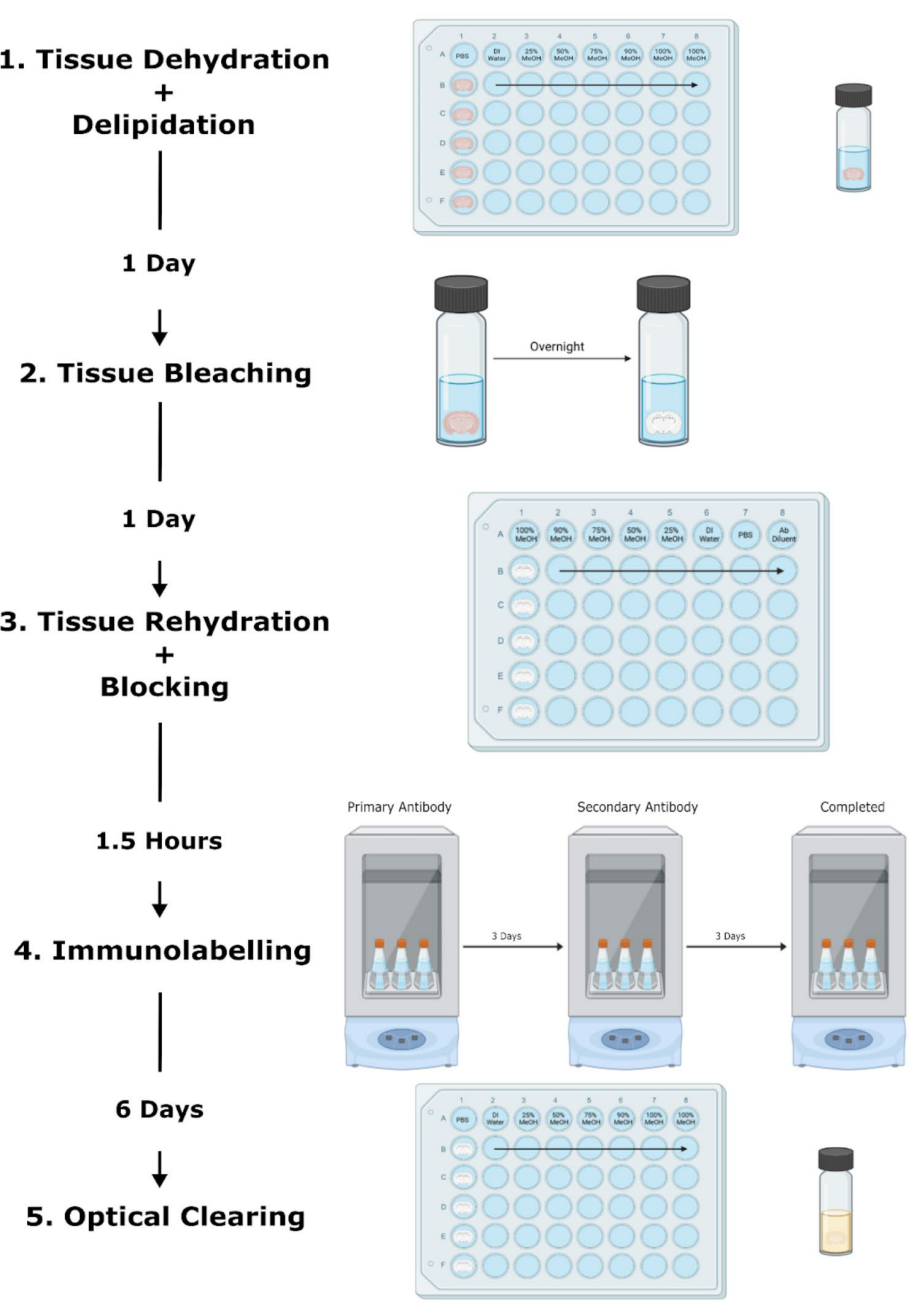
Schematic representation of ABSOC workflow including time-lapsed between steps. Methanol – MeOH; deionised water – DI

The ABSOC protocol is described in Fig. 1, which illustrates the steps below to optically clear and immunolabel calretinin in 1 mm brain slices:

1. **Dehydration and delipidation.** These steps were performed on a shaker at RT. Brain slices were gradually incubated in 1 ml solutions – PBS, deionized water (dH_2_O), 25%, 50%, 75%, 90% methanol (diluted in dH_2_O), and 2 × 100% methanol (+ 0.3% triethylamine (TEA)) for 10 min per solution, in order to dehydrate the slices. Gradual changes in concentrations were used to minimize tissue shrinkage. Following dehydration, the slices were moved into glass amber vials and incubated in 2 ml 66% dichloromethane (DCM – prepared in 100% methanol) overnight to delipidate the tissue. DCM is hazardous and corrosive to certain plastics, reagents needs to be prepared in a fume hood and stored in glass bottles.
2. **Tissue Bleaching.** Brain slices were washed twice in 1 ml 100% methanol + 0.3% TEA, and then incubated with 2 ml 5% hydrogen peroxidase (prepared in 100% methanol + 0.3% TEA) overnight on a shaker at RT.
3. **Tissue Rehydration and Blocking.** These steps were performed on a shaker at RT. Brain slices were rehydrated through gradually decreasing concentrations of 1 ml methanol (100% (+ 0.3% TEA), 90%, 75%, 50%, 25%, dH_2_O, PBS) for 5 min per solution. The tissue was then blocked using an antibody diluent (PBS + 0.05% sodium azide + 1.5% Normal Goat Serum + 0.3% Triton-X) for 1 h to minimize non-specific antibody binding.
4. **Immunolabelling.** This step was performed inside a shaking incubator at 37^o^C. Brain slices were incubated for 3 days in 2 ml primary antibody solutions of 1:5000 (0.55 mg/ml) anti-calretinin antibody (prepared in antibody diluent). Following primary antibody incubation, brain slices were washed 4 × (30 s, 1 h, 1 h, and 15 min) in 1 ml antibody diluent to stop primary antibody binding before incubation with 2 ml 1:1000 anti-Rabbit AlexaFluor633 secondary antibodies (prepared in antibody diluent) for 3 days. After secondary antibody incubation, brain slices were washed 4 × (30 s, 1 h, 1 h, and 15 min) in 1 ml antibody diluent to remove secondary antibody.
5. **Optical Clearing.** After immunolabelling, brain slices were dehydrated using 1 ml methanol (PBS, dH_2_O, 25%, 50%, 75%, 90%, and 2 × 100% (+ TEA) methanol) on a shaker for 5 min per solution at RT. Following dehydration, slices were washed once in 1 ml 1-part benzyl alcohol and 2-part benzyl benzoate (BABB) for 10 min and then incubated in 1 ml BABB until imaging. BABB has a similar refractive index as dehydrated protein so at this stage brain slices were optically clear inside the BABB solution.

### Image acquisition

**Fig. 2:**
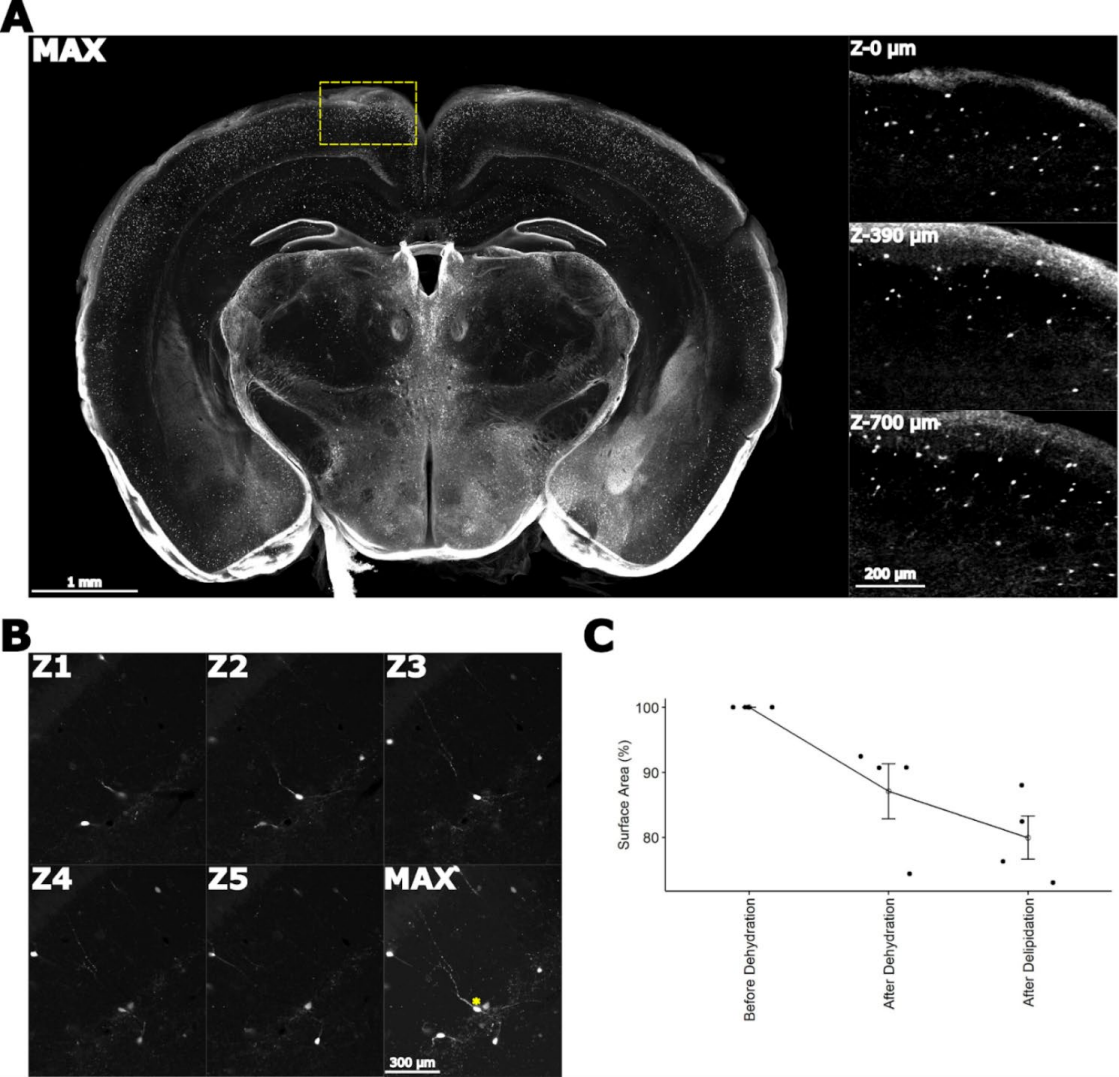
Calretinin + immunolabelling in an ABSOC cleared 1 mm thick brain slice. (**A**) Maximum intensity projection (MAX) of calretinin-stained brain slice imaged using a x10 air objective (2.9 × 2.9 × 5.0 µm^3^), with higher resolution images of the cortex (yellow box in MAX) through the z-plane taken with an x20 oil objective (1.4 × 1.4 × 5.0 µm^3^). (**B**) 3D reconstruction of single calretinin + cells taken from 445–470 μm distance in the 700 μm z-stack of the x20 image. Images from z-stack (Z1-Z5 panels) are separated by 5 μm step-size in each increment, a maximum projection image of single cells is illustrated in MAX, and, * is identifying a cell body that has been completely reconstructed in the images. (**C**) Quantitative analysis of tissue shrinkage during dehydration and delipidation steps demonstrated ~ 20% shrinkage in surface area occurs in 1 mm sections during ABSOC. n = 4 sections, and data presented as mean ± SEM

Following optically clearing and immunolabeling of the tissue slices, the sections were mounted on glass microscope slides, fitted inside an in-house made 800 μm silicone spacer with fresh BABB solution. Given that the tissue is optically cleared, image acquisition on a confocal/epifluorescence microscope is limited by the working distance of the objective. Accurate high resolution acquisition throughout the z stack should utilise an oil-immersion objective, which guarantees a consistent refractive index throughout the lightpath (of oil 1.52, glass 1.52, tissue/BABB 1.56). For low resolution acquisition, an air objective with long working distance delivers acceptable results (Fig. 2A).

Calretinin is primarily expressed by interneurons in the cerebral cortex and immature granule cells within the dentate gyrus of the hippocampal formation (Fig. 2A). We observed even and consistent calretinin cell body labelling in a 1 mm mouse brain coronal section imaged using a x10 objective on a Leica SP8 confocal microscope at 2.89 µm^2^ pixel resolution and 7 μm z-step size (Fig. 2A). Using a higher magnification oil-immersion objective at x20, we can observe complete 3D reconstructions of single cells (Fig. 2B).

We observed approximately 20% shrinkage in the surface area of the tissue (Fig. 2C), an unavoidable consequence of the dehydration and organic mounting media.

Additionally, we found ABSOC to be compatible in other mouse brain regions such as the medial prefrontal cortex (Supplementary Fig. 1) and with other non-neuronal antibody markers like GFAP, IBA1, and S100B (Supplementary Fig. 2).

## Discussion

ABSOC is a simple and affordable method to clear and immunolabel brain slices without the need for specialised equipment or expert training. Here, we describe the ABSOC method and we show results from immunolabeling calretinin positive cells in 1 mm thick mouse brain slices to illustrate the effectiveness of this technique. We believe this method will enable biologists to use tissue blocks for immunolabeling, thus giving more accurate 3D representations of cellular environments than is possible from thin sections alone.

Using ABSOC, we immunolabelled 1 mm thick mouse brain slices with calretinin and showed positive cells throughout the depth of the tissue, and importantly, we were able to further interrogate our results by 3D reconstruction of single cells. The advantage of this technique lies in its ability to sample large tissue blocks, and reconstruct structures and cells in 3D environments, making it possible to analyse structures free from 2D biases.

Additionally, a key benefit of the ABSOC method is its ability to retain tissue integrity. The significant level of fixation conveyed by 24 h in 4% formaldehyde at room temperature provides strong crosslinked tissue preparations. Combined with the rigidity induced by dehydration, tissue integrity and therefore morphology is excellent in ABSOC tissue. Antigenicity tends to be impacted by solvent treatments, and has been shown to be dependent on pH [12]. ABSOC utilises triethylamine in solvent washes to reduce the impact of solvent treatment during delipidation on tissue antigenicity. Here we show excellent preservation of antigenicity to calretinin, consistent with this protection.

Poor penetration of antibodies is commonly described in histological assessment of large tissue blocks, and various barometric and electrophoresis tools have been designed to overcome this issue [8, 13]. In ABSOC, we address this issue by simply incubating tissue blocks with low concentration of the antibody coupled with high volume of the diluent - this allows sufficient antibody to permeate through the tissue, evident through the strong cell body signal we observed throughout our tissue. Calretinin antibodies label strongly the soma and proximal dendrites of calretinin-positive cells, but weakly the peripheral thin processes, which reflects the strong antibody signal we detect in the periphery of our calretinin immunolabelled brain slices [14–16]. Strong antibody labelling in the periphery of the tissue blocks was not observed using non-neuronal markers such as GFAP, IBA1, and S100B – suggesting this staining profile is unique to calretinin expression (Supplementary Fig. 2). Using ABSOC results in higher fluorescent signal on the edges of the tissue compared to the middle, but maintains labelling so that we are able to observe the cells throughout the entire tissue block (Fig. 2). Additionally, we recommend researchers who plan on analysing cell counts to add guard zones to their z-stacks, as non-specific labelling at the surfaces of the tissue could potentially skew their results - this is a practice commonly used in stereology [17].

ABSOC can be readily adapted for laboratories using mouse brain immunofluorescence to understand distribution and morphology of cells to move from the routine use of sampling thin tissue sections, to larger tissue blocks, facilitating 3D reconstructions of structures, and hence more unbiased analysis in their experiments.

## Limitations

We have observed ~ 20% tissue shrinkage during clearing due to delipidation and dehydration, similar that observed in iDISCO [5]. This is an issue for white matter or gross anatomical analysis of tissue regions, although observation of cellular morphology or cell numbers is unaffected [5]. Other non-solvent based clearing methods also induce tissue deformation such as tissue expansion [3, 18]. Future work needs to address these alterations to tissue volume during clearing.

Imaging of endogenous fluorescent proteins is not possible using ABSOC – all endogenous proteins are reportedly quenched by the use of methanol, DCM, and BABB [4]. Several modified iDISCO methodologies such as 3DISCO [4], fDISCO [19], sDISCO [20] have been adapted to allow for endogenous expression of fluorescent protein, largely by replacing methanol with tetrahydrofuran (THF) during tissue dehydration and BABB with dibenzyl ether during refractive index matching. Whilst THF has the improved ability to maintain endogenous fluorescence, it induces more tissue shrinkage (~ 50% volume) compared to using methanol for dehydration [21]. Exploring the use of these solvents in antibody labelling and maintaining endogenous fluorescence with minimal changes to tissue volume will be of huge benefit to the scientific community.

### Electronic supplementary material

Below is the link to the electronic supplementary material.

Supplementary Material 1

## Data Availability

Further information and data requests should be directed to the lead contact: Phillip Muza (p.muza@ucl.ac.uk) or corresponding author: Steven West (s.west@ucl.ac.uk).

## Abbreviations

ABSOC: Affordable Brain Slice Optical Clearing
IHC: Immunohistochemistry
RT: Room temperature
TEA: Triethylamine
DCM: Dichloromethane
BABB: Benzyl alcohol, benzyl benzoate
MAX: Maximum intensity projection
THF: Tetrahydrofuran
PBS: Phosphate buffer solution
WT: Wild-type
