# Supplementary material for: Affordable optical clearing and immunolabelling in mouse brain slices
Source: BMC Res Notes. 2023 Sep 30;16:246. doi: 10.1186/s13104-023-06511-y (PMC10543858; doi:10.1186/s13104-023-06511-y)
Supplement: Supplementary file 1 — Supplementary Material 1 [file 13104_2023_6511_MOESM1_ESM.docx]

| 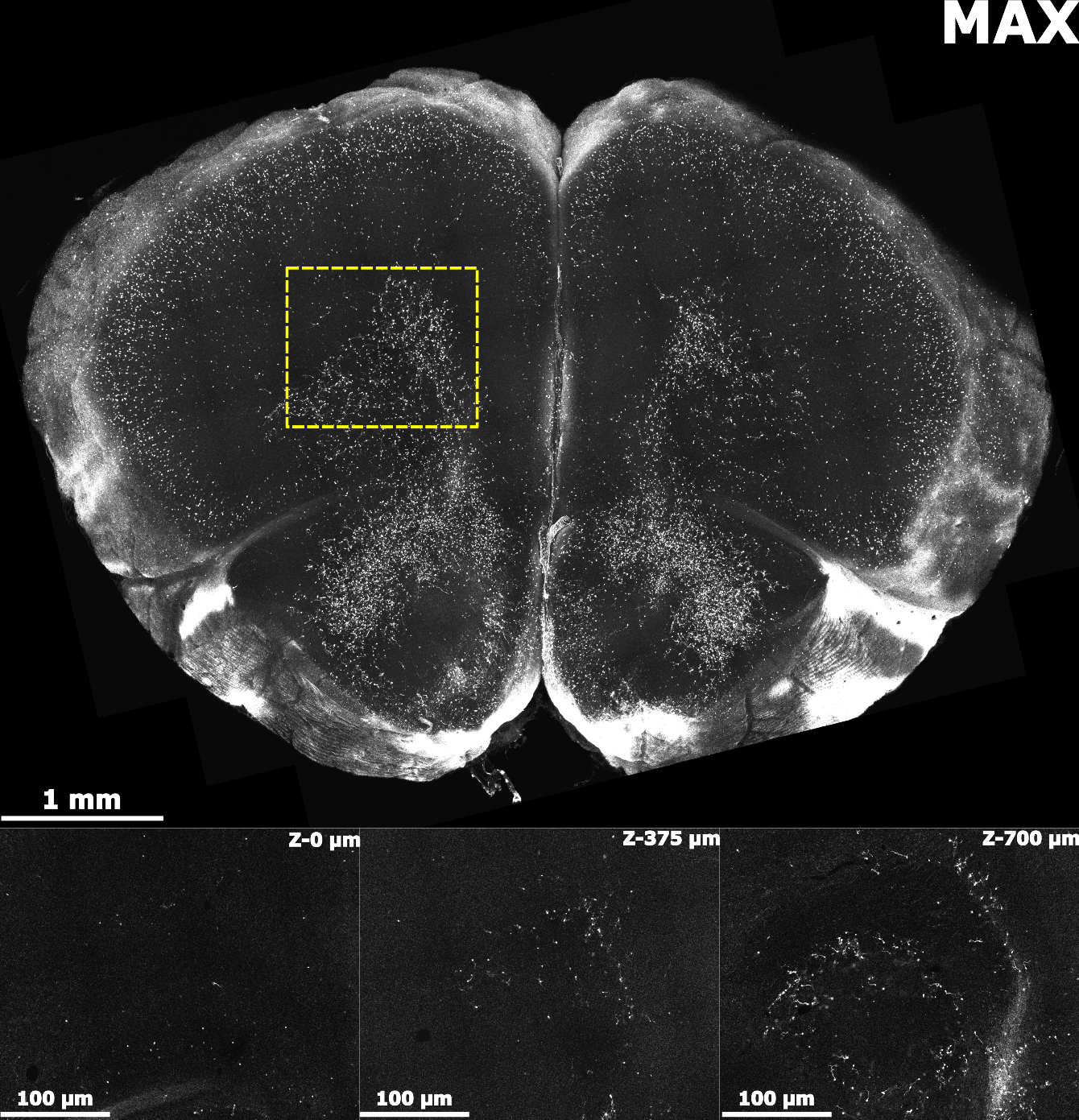 |
| --- |
| **Supplementary Figure 1. Calretinin+ immunolabelling in a 1 mm coronal brain slice from a C57BL/6J mouse sectioned through the medial prefrontal cortex.** Maximum projection image of calretinin-stained slice imaged using a x20 IMM objective (1.44 x 1.44 x 25 μm^3^) and images of the genu of the corpus callosum (yellow box in MAX) through the z-plane are also provided in the bottom panel. |

| 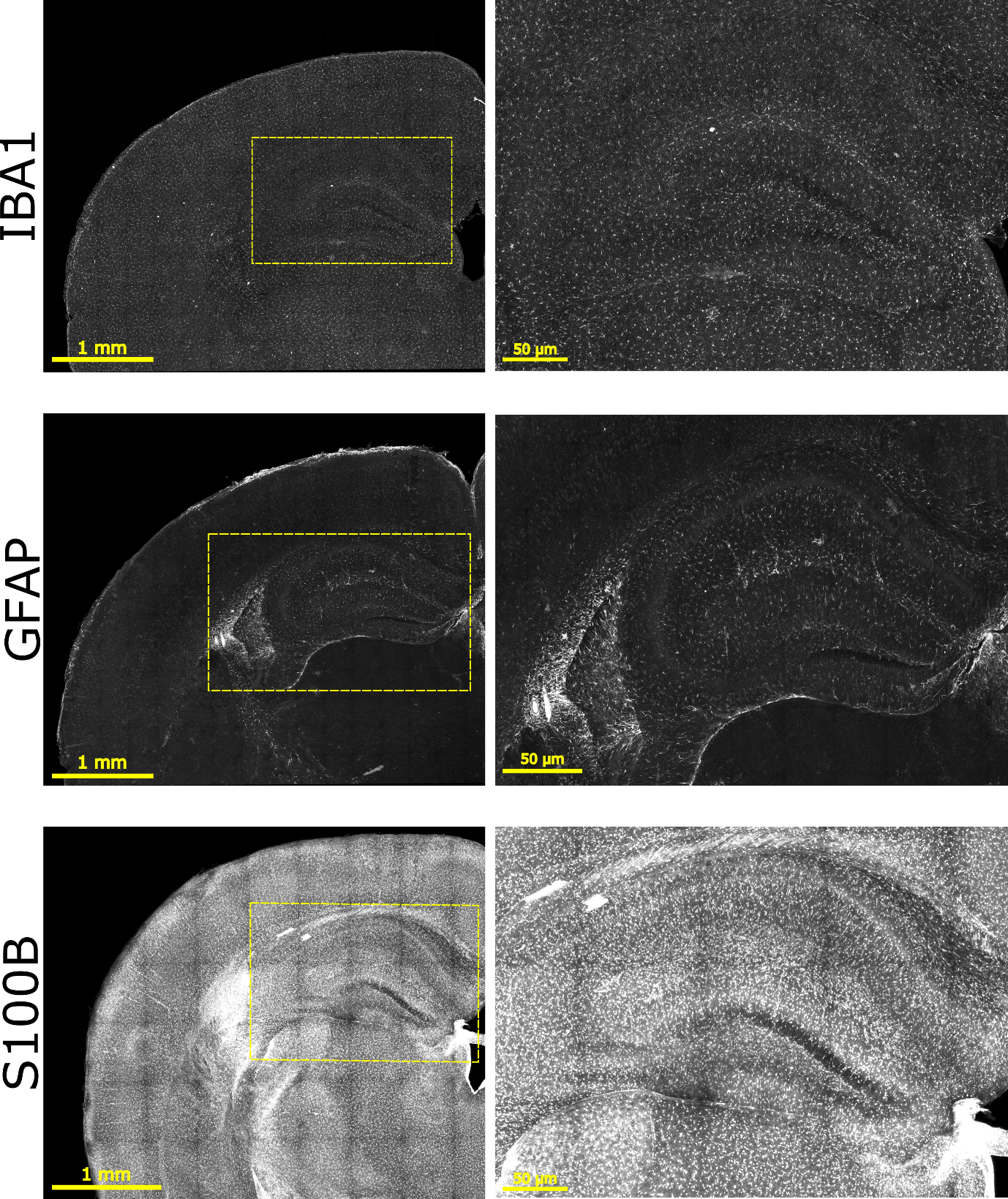 |
| --- |
| **Supplementary Figure 2. Immunolabelling of non-neuronal cells expressing IBA1, GFAP, or S100B, using ABSOC in 400 µm thick coronal brain slices sectioned through the dorsal hippocampus of C57BL/6J mice.** Images were acquired using a x40 IMM objective (0.6923 x 0.6923 x 4 um^3^), and close up images of the hippocampus (yellow box in MAX) are provided on the right panel for each protein immunolabelled. |
